# Evaluation of screening tests for autoimmune gastritis in histopathologically confirmed Japanese patients, and re-evaluation of histopathological classification

**DOI:** 10.1186/s12876-022-02251-8

**Published:** 2022-04-11

**Authors:** Yasuhiro Wada, Shigemi Nakajima, Naoko Mori, Shizuki Takemura, Rena Chatani, Mariko Ohara, Makoto Fujii, Hiroshi Hasegawa, Kiyoyuki Hayafuji, Ryoji Kushima, Kazunari Murakami

**Affiliations:** 1 Consortium for Community Medicine, Shiga University of Medical Science, Otsu, Shiga Japan; 2 Department of General Medicine, Japan Community Healthcare Organization (JCHO) Shiga Hospital, Consortium for Community Medicine, Shiga University of Medical Science, 16-1, Fujimidai, Otsu, Shiga 520-0846 Japan; 3 Department of Gastroenterology, Japan Community Healthcare Organization (JCHO) Shiga Hospital, Otsu, Shiga Japan; 4 Department of Gastroenterology, Faculty of Medicine, Oita University, Yufu, Oita Japan; 5 Department of Pathology, Omi Medical Center, Kusatsu, Shiga Japan; 6 Department of Pathology, Shiga University of Medical Science, Otsu, Shiga Japan

**Keywords:** Anti-parietal cell antibody, Gastrin, Corpus atrophy, Pepsinogen, Vitamin B_12_

## Abstract

**Background:**

The aims of the present study are to evaluate non-invasive screening tests for autoimmune gastritis (AIG) and re-evaluate histopathological classification.

**Methods:**

We screened candidates of AIG in JCHO Shiga Hospital between May 2012 and January 2020. The screening criteria were as follows: endoscopic O-p atrophy with Updated Kimura–Takemoto classification, 3 + pepsinogen (PG) test, low serum vitamin B_12_ or elevated serum gastrin with positive anti-parietal cell (PC) or intrinsic factor antibodies. We evaluated the screening criteria in the patients who were histopathologically confirmed as AIG, and re-evaluated histopathological staging in clinical aspects.

**Results:**

Twenty-two of 28 (78.6%) patients who met the screening criteria were histopathologically confirmed as AIG. Common clinical findings in the AIG patients were 10 × or greater anti-PC antibody, elevated serum gastrin greater than 172 pg/mL and endoscopic atrophy O-1 or greater. The areas under the curve of PG I, PG II and PG I/II ratio were 0.81, 0.29 and 0.98, respectively. Among histopathologically confirmed AIG patients, 4 and 18 patients were histopathologically classified into florid and end stages, respectively, while no patients into early stage. We could not find a significant difference between florid and end stages in the screening items studied.

**Conclusions:**

Florid and end stages in histopathological classification are both advanced-stage AIG in clinical aspects. Our screening criteria without biopsy are applicable to screen clinically-advanced AIG with 78.6% positive predictive value. PG I and PG I/II ratio may be useful to screen AIG. However, we may need other criteria to screen early stage of AIG.

## Background

Autoimmune gastritis (AIG) was summarized in 1973 by Strickland and Mackey as diseases with fundic gland atrophy, decreased gastric acid secretion, positive anti-parietal cell (PC) antibody or anti-intrinsic factor (IF) antibody and elevated serum gastrin often accompanied with pernicious anemia [1]. AIG had been considered uncommon in Asian countries until recently [2, 3]. However, some studies in Japan reported that the actual prevalence of AIG would be higher than believed. Notsu et al. reported that the prevalence of AIG was at least 0.49% in 6739 subjects with endoscopic upper GI screening [4]. On the other hand, much more undiagnosed candidates of AIG were found in Western countries [5, 6]. In Germany, anti-PC antibody is present in 19.5% of the 9684 individuals undergone health checkups [6]. To know the precise prevalence of AIG, we need large number of subjects to be screened.

Currently, the final diagnosis of AIG often depends on histopathological findings in biopsy specimens which are taken with an invasive procedure. Before taking biopsy specimens we need clinical criteria to screen AIG without invasive procedures. Serum gastrin may be a good tool to screen AIG, but we do not know whether it is enough. To screen AIG, fundic gland atrophy is essential. Endoscopic evaluation of atrophy is established by Kimura and Takemoto in 1968, and recently it has been updated [7, 8]. Serum pepsinogen (PG) test has been used as biomarkers of gastric atrophy and cancer risk [9]. Thus endoscopic evaluation of gastric atrophy and PG test may be useful to screen AIG. In addition, serum vitamin B_12_ may be a good tool to screen IF deficiency in AIG. We have recently proposed screening criteria of AIG including these tests to screen more patients with AIG [10], so we have to clarify whether the criteria are appropriate in histopathologically confirmed AIG. Histopathologically, AIG have been classified into early, florid and end stages [11–13], but the clinical differences between these stages are not clearly elucidated.

In the present study, we evaluated our screening criteria for AIG in histopathological aspects and re-evaluated the histopathological classification in clinical aspects.

## Methods

### Subjects

We collected medical information of the candidates who were clinically suspected of AIG in Japan Community Healthcare Organization (JCHO) Shiga Hospital between May 2012 and January 2020. The screening criteria for candidates were as in the followings: marked endoscopic corpus atrophy (O-p in Updated Kimura–Takemoto classification) [7], severely positive (3 +) PG test, low serum vitamin B_12_ or elevated serum gastrin, accompanied with positive anti-PC or IF antibodies [10]. Among them, those who were taken biopsy specimens were histopathologically evaluated as described below. Because patients who have renal dysfunction shows elevated serum gastrin and pepsinogens [14, 15], we excluded patients with abnormal renal function tests.

### Tests and the procedures

Anti-PC antibody and anti-IF antibody were measured with anti-mitochondrial antibody/anti-smooth muscle antibody FA (Fujirebio, Tokyo, Japan) and Beckman Coulter ACCESS intrinsic factor Ab (Beckman Coulter, Brea, USA), respectively. Since the above tests were not covered with medical insurance in Japan, we could not examine all the suspected patients. If the patient accepted to pay, we tested anti-PC antibody first, and then tested anti-IF antibody if the first one was negative and the patient accepted to pay for the next one. Those who did not accept the tests were not included in the candidates. Endoscopic gastric atrophy was evaluated with Updated Kimura–Takemoto classification [7, 8]. Briefly, endoscopic atrophic border was classified into 8 grades: C-0, no atrophy; C-1, closed border in the antrum; C-2, closed border in the distal corpus; C-3, closed border in the proximal corpus; O-1, open border in the lessor curvature; O-2, open border between lessor and greater curvatures; O-3, open border in the greater curvature; O-p, pan atrophy in the corpus without atrophic border. Serum PG was measured with ARCHITECT Pepsinogen I and II (Abbott Japan, Tokyo, Japan). According to Miki and Urita [9], all patients were classified into four groups: severely positive (3 +), serum PG I ≤ 30 ng/ml and PG I/II ratio ≤ 2.0; moderately positive (2 +), serum PG I ≤ 50 ng/ml and PG I/II ratio ≤ 3.0; mildly positive (+), serum PG I ≤ 70 ng/ml and PG I/II ratio ≤ 3.0; and negative (−), the remainings. Because PG tests were not covered by medical insurance in Japan, we did not test them in all the candidates. Serum gastrin and vitamin B_12_ were measured with Gastrin RIA Kit II (Fujirebio) and Beckman Coulter ACCESS B_12_ (Beckman Coulter), respectively. Values ​​higher than 172 pg/mL for gastrin or values lower than 180 pg/mL for vitamin B_12_ were defined high or low, respectively, according to the manufacturer’s instruction.

### Histopathological diagnosis

For histopathological evaluation, biopsy specimens were endoscopically taken from the greater curvature of the middle corpus and the antrum. Biopsy specimens were immediately fixed in 10% neutral buffered formalin for 24 h and embedded in paraffin. Samples were sliced into 3 μm-thick sections and stained with hematoxylin and eosin (H&E). We histopathologically confirmed AIG by observing the status of inflammatory cell infiltration and atrophy of lamina propria in the middle corpus mucosa, while comparing the findings of antral mucosa. AIG was divided into three stages. The early stage consisted of patchily decreased fundic glands with diffuse or multifocal dense basal-predominant lymphoplasmacytic infiltration. The florid stage consisted of marked decrease of fundic glands with lymphoplasmacytic infiltration. The end stage consisted of complete parietal cell loss with minimal inflammatory cell infiltration [11–13]. Atrophy of fundic glands included marked decrease or loss of fundic glands with or without pyloric or intestinal metaplasia. Enterochromaffin cell-like (ECL) cell hyperplasia in the corpus and gastrin cell hyperplasia in the antrum were supporting findings to diagnose AIG. Histopathological diagnosis was performed by two pathologists and consensus was made if the diagnoses were different.

### *Helicobacter pylori* (*H. pylori)* infection

In order to confirm the infection status of *H. pylori*, history about the past *H. pylori* tests and therapies was collected from the medical records. *H. pylori* tests included serum *H. pylori* antibody test (HpAb) (E-plate Eiken *H. pylori* antibody II, Eiken Kagaku, Tochigi, Japan), *H. pylori* stool antigen test (HpSA) (Meridian HpSA ELISA II, Fujirebio), urea breath test (UBT) (Ubit Tablets 100 mg, Otsuka Pharmaceutical, Tokyo, Japan; POCone, Otsuka Electronics, Osaka, Japan), histopathology (H&E staining and Giemsa staining) and culture for *H. pylori*. Serum anti-*H. pylori* was tentatively judged negative when the ELISA value was below 10.0 U/mL according to manufacturer’s instruction. HpSA was tentatively judged positive with the value 0.120 or more and negative with the values less than 0.100. UBT was tentatively judged negative with the Δ^13^CO2 (UBT value) less than 2.0‰ and positive with the value 5.0‰ or more. Patients with clear evidence of current *H. pylori* infection with pathology or culture were determined "currently infected". Those showing no evidence of infection by all the tests performed were diagnosed "not infected". Those who had past eradication therapy were included in “past infected”. Other patients were diagnosed either of the above three status from the combination of multiple tests and their chronological changes.

### Statistical analysis

To evaluate serum gastrin, PGs and vitamin B_12_ among AIG stages, *t*-test was indicated. To evaluate endoscopic corpus atrophy, *chi*-square test was indicated. SPSS (Stats Guild Inc. Chiba, Japan) was used in each analysis and a value of *P* < 0.05 was considered significant.

## Results

We found 29 candidates who met the screening criteria and were taken biopsy specimens during the study period. One patient was excluded from the study because the tissue from the corpus was too small to evaluate atrophy of fundic glands. Of the remaining 28 patients, 22 patients (78.6%, 95% confidence interval 59.0–91.7) were histopathologically confirmed AIG and studied in the following sections.

**Fig. 1:**
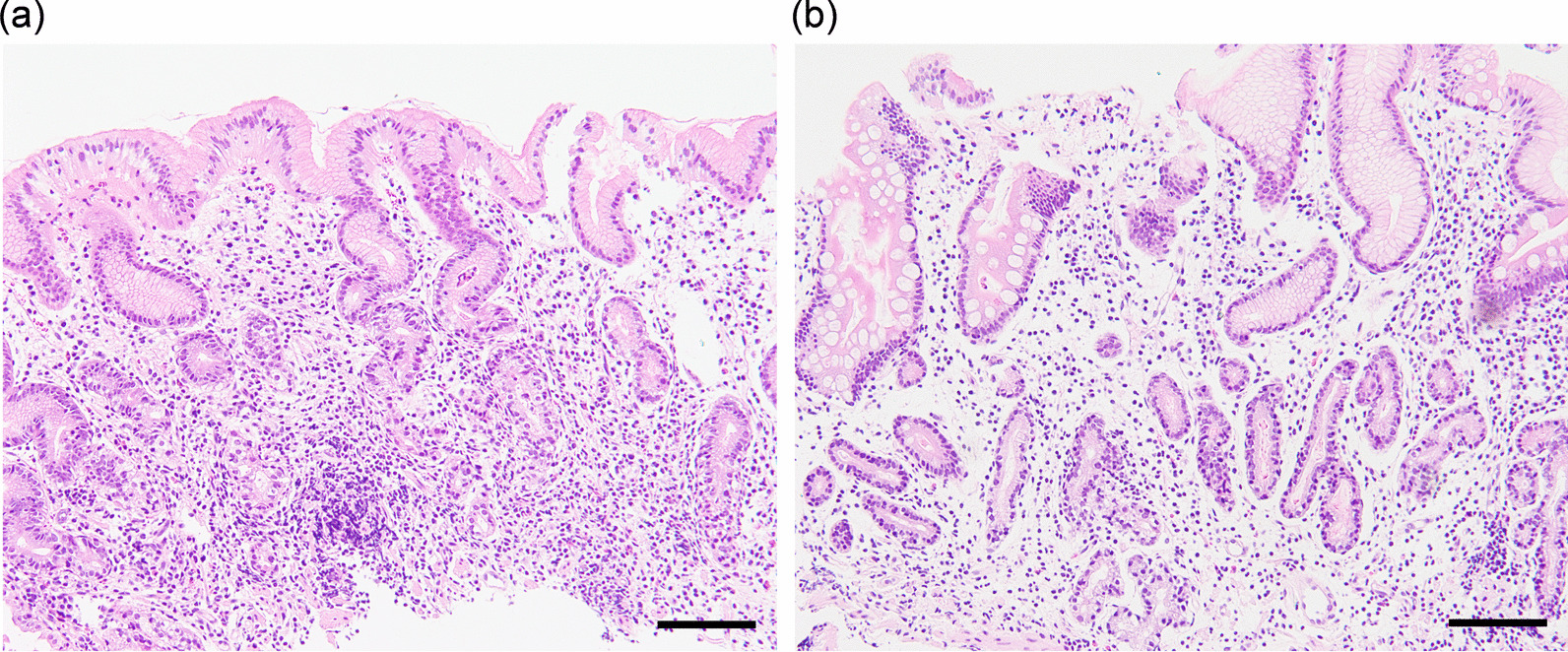
Light microscopic pictures of florid and end stages (H&E stain). The florid stage consisted of marked decrease of fundic glands with lymphoplasmacytic infiltration predominantly in the deeper part of the mucosa (**a**). The end stage consisted of complete loss of fundic glands with minimal inflammatory cell infiltration (**b**). Scale bar: 200 µm

Of the 22 AIG patients, 7 were men and 15 were women. Age ranged 43–81 years (mean ± SD = 65.2 ± 10.8 years). Two patients used vitamin B_12_-containing agents. There were no patients using proton pump inhibitor (PPI). Currently *H. pylori*-infected patients were not found in any of the 22 AIG patients. On the other hand, 12 (54.5%) and 10 patients (45.5%) were diagnosed as past and not infected, respectively. However, the details of the previous eradication therapies were unknown in 5 of the former 12 patients. The duration between the previous eradication therapy and the diagnosis of AIG was 1 year in 3 patients, 2 years in 1 patient, 3 years in 2 patients and 4 years in 1 patient (Table 1). Four patients (18.2%) and 18 patients (81.8%) were histopathologically classified into florid and end stages of AIG, respectively (Table 2, Fig. 1). No patients were histopathologically classified into early stage of AIG.

**Table 1:** Duration between *H. pylori* eradication therapy and the diagnosis of AIG

Years between eradication and AIG diagnosis	Number of patients
1	3
2	1
3	2
4	1
Unknown	5
Total	12

*H. pylori*, *Helicobacter pylori*; AIG, autoimmune gastritis

**Table 2:** List of 28 AIG candidates

No	Sex	Age	AIG stage	PC antibody	IF antibody	positive items	Gastrin (pg/mL)	Endoscopic corpus atrophy	PG I (ng/mL)	PG II (ng/mL)	PG I/II ratio	PG test	Vitamin B_12_ (pg/mL)	*H. pylori* infection	Comments
1	M	63	Florid	160	Positive	1	1250	O-3	64.8	18.5	3.5	−	1060	Past	Using B_12_-containing agents
2	F	68	Florid	40	NE	3	1800	O-p	4.2	7.9	0.5	3 +	188	Past	
3	F	53	Florid	80	NE	4	1400	O-p	11.4	9.4	1.2	3 +	97	Past	
4	F	60	Florid	80	NE	4	2180	O-p	10.4	11.7	0.9	3 +	157	Past	
5	F	66	End	40	NE	2	972	O-p	NE	NE	NE	NE	183	Past	
6	M	43	End	80	NE	2	1170	O-1	NE	NE	NE	NE	86	No	
7	F	77	End	80	NE	2	1110	O-p	71.9	39.2	1.8	−	247	Past	
8	F	70	End	10	NE	3	851	O-p	33.3	25.9	1.3	2 +	71	Past	
9	F	44	End	20	NE	3	5300	O-p	3.1	5.2	0.6	3 +	270	No	
10	F	81	End	40	NE	3	4020	O-p	4.4	4.4	1	3 +	236	No	
11	F	81	End	80	NE	3	696	O-p	6.9	13.5	0.5	3 +	320	Past	
12	M	53	End	160	NE	3	720	O-p	7.1	7.3	1	3 +	180	Past	
13	F	66	End	160	NE	3	2250	O-p	8.4	6.3	1.3	3 +	435	Past	Using B_12_-containing agents
14	M	78	End	10	NE	4	708	O-p	2.5	3.7	0.7	3 +	56	No	
15	F	60	End	20	NE	4	5000	O-p	9.1	10.9	0.8	3 +	105	No	
16	M	67	End	20	NE	4	2170	O-p	2.5	5.3	0.5	3 +	50	Past	
17	F	70	End	20	NE	4	5017	O-p	3.7	11.9	0.3	3 +	70	Past	
18	M	71	End	20	NE	4	4054	O-p	8.8	4.9	1.8	3 +	51	No	
19	F	71	End	20	NE	4	690	O-p	3.8	4.6	0.8	3 +	52	No	
20	F	56	End	40	NE	4	2493	O-p	2.7	7.9	0.3	3 +	92	No	
21	F	79	End	40	NE	4	3350	O-p	3.1	11.1	0.3	3 +	116	No	
22	M	57	End	160	NE	4	1600	O-p	4.6	7.9	0.6	3 +	122	No	
23	M	73	Not AIG	10	NE	1	307	O-3	17.5	7.4	2.4	2 +	330	Past	
24	F	68	Not AIG	20	NE	1	93	O-p	70.6	11.2	6.3	−	377	Past	
25	F	65	Not AIG	40	NE	1	121	O-p	22.9	4.7	4.9	−	479	Past	
26	M	67	Not AIG	80	NE	1	72	O-3	23.1	4.9	4.7	−	178	Past	
27	F	73	Not AIG	10	NE	2	300	O-p	20.7	6.1	3.4	−	623	Past	
28	M	80	Not AIG	10	NE	2	195	O-p	5.3	2.3	2.3	2 +	604	Past	Using B_12_-containing agents
							COV = 172						COV = 180		

Endoscopic corpus atrophy was evaluated with Updated Kimura–Takemoto classification

AIG, autoimmune gastritis; PC antibody, anti-parietal cell antibody; IF antibody, anti-intrinsic factor antibody; PG, pepsinogen; *H. pylori*, *Helicobacter pylori*; NE, not examined; COV, cut-off value; B_12_, vitamin B_12_

Positive items, the number of positive items among following 4 tests: elevated serum gastrin, endoscopic O-p atrophy, 3 + PG test and low serum vitamin B_12_

In 22 AIG patients, anti-PC antibody ranged from 10 × to more than 160x (10 × in 2 patients, 20 × in 6 patients, 40 × in 5 patients, 80 × in 5 patients, 160 × or greater in 4 patients). One patient was also positive for anti-IF antibody.

**Fig. 2:**
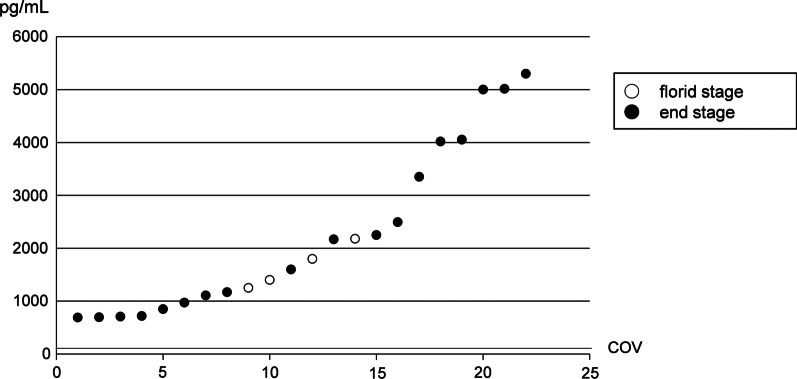
Distribution of serum gastrin of all AIG patients (N = 22). COV; cut-off value (172 pg/mL). The numbers in the horizontal axis were not patient’s numbers in Table 2. Patients are ordered by the values of serum gastrin. All 22 patients showed high serum gastrin. Four patients were included in florid stage (○), and 18 patients were included in end stage (●)

Serum gastrin was measured in all 22 AIG patients. In these AIG patients, gastrin ranged from 690 to 5300 (mean ± SD = 2218.2 ± 1509.6) pg/mL and all these values exceeded the normal range (172 pg/mL). Of these 22 patients, 4 and 18 patients were included in the florid and end stages, respectively. Serum gastrin was not statistically different between histopathological stages of AIG (florid stage: mean ± SD = 1657.5 ± 362.5 pg/mL; end stage: 2342.8 ± 1634.3 pg/mL; *P* = 0.14, *t*-test), but those who showed higher serum gastrin than 3000 pg/mL were all in end stage (Fig. 2).

**Fig. 3:**
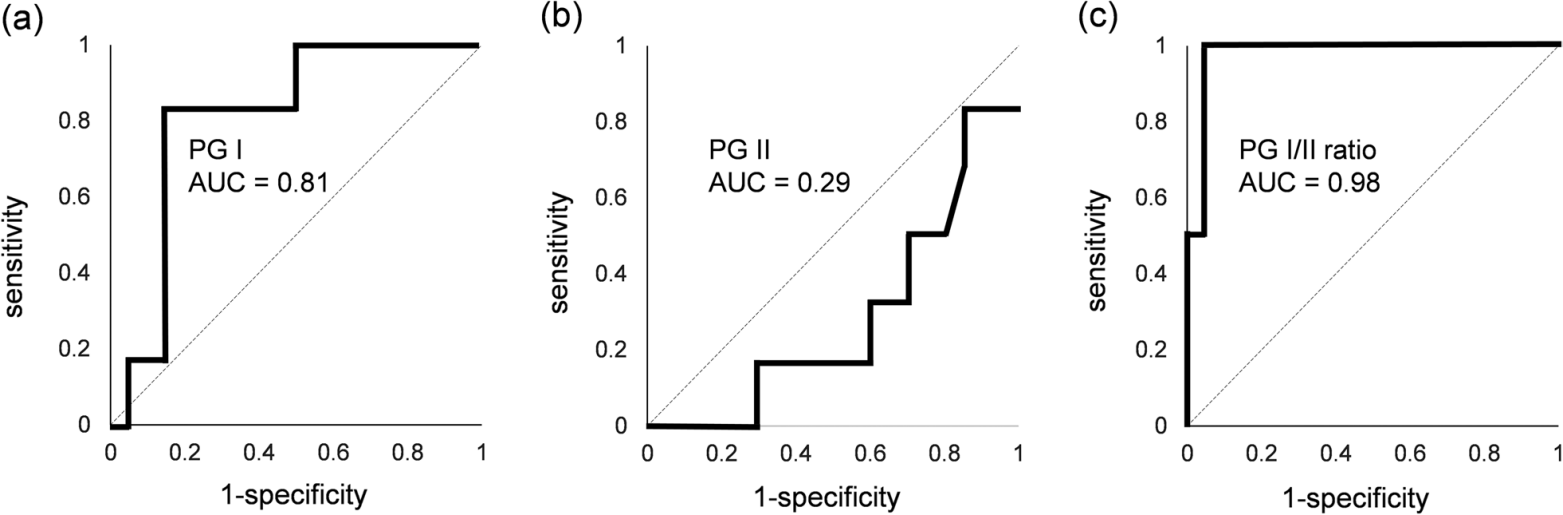
Receiver operating characteristic (ROC) curves of PG I (**a**), PG II (**b**) and PG I/II ratio (**c**). PG; pepsinogen, AUC; area under the curve. AUC of PG I, PG II and PG I/II ratio were 0.81, 0.29 and 0.98, respectively. According to ROC curve analyses, the optimal cut-off values of PG I and PG I/II ratio were 14.5 ng/mL (sensitivity = 0.83, specificity = 0.85) and 2.1 (sensitivity = 1.00, specificity = 0.95), respectively

**Fig. 4:**
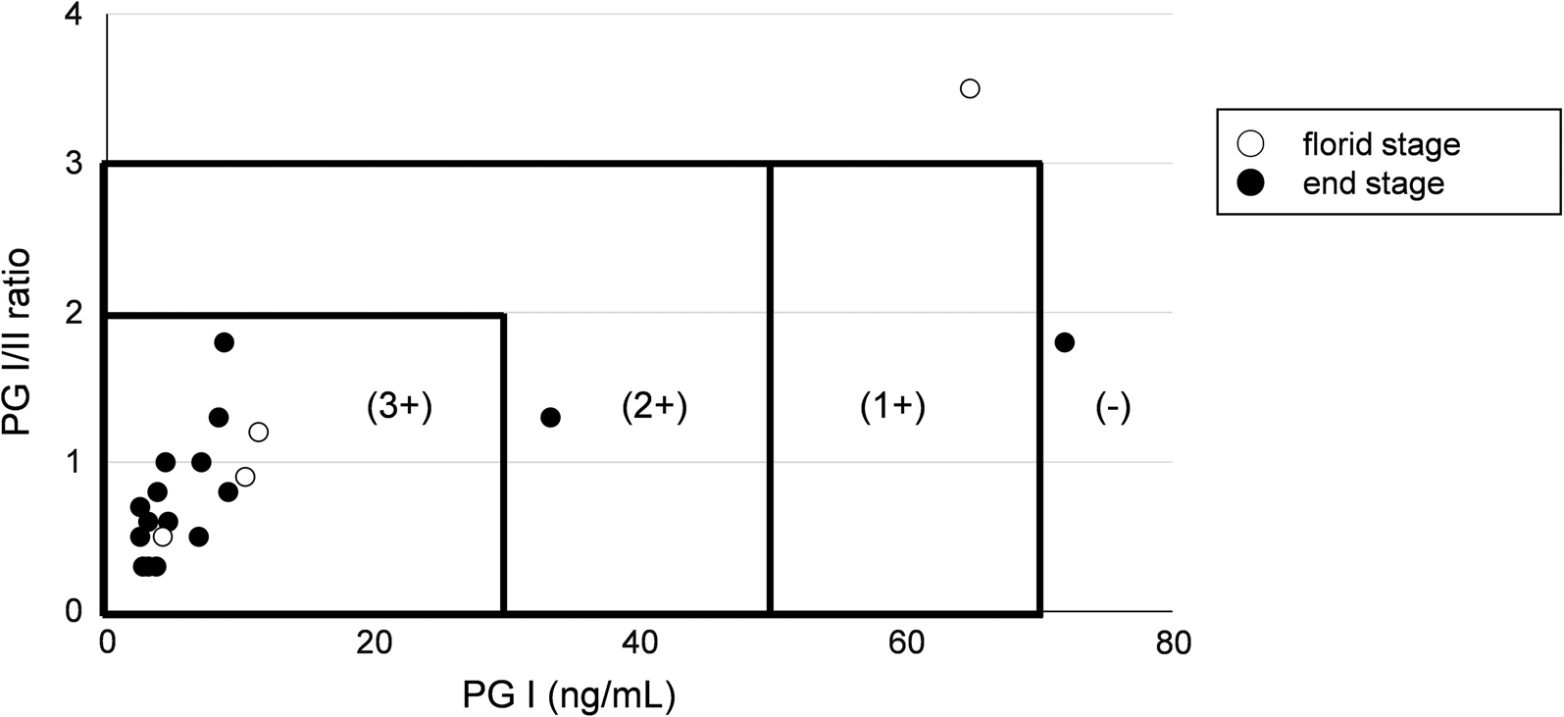
Distribution of PG test of 20 AIG patients (N = 20). PG; pepsinogen. PG test classification revealed 3 + in 17 (85%), 2 + in 1 (5%), 1 + in 0 (0%) and negative in 2 patients (10%). Four patients were included in florid stage (○), and 16 patients were included in end stage (●)

We analyzed cut-off values for PGs with receiver operating characteristic (ROC) curves. The areas under the curve (AUC) of PG I, PG II and PG I/II ratio were 0.81, 0.29 and 0.98, respectively (Fig. 3), indicating that PG I and PG I/II ratio were suitable for making cut-off values. The optimal cut-off values for PG I and PG I/II ratio were suggested as 14.5 ng/mL (sensitivity = 0.83, specificity = 0.85) and 2.1 (sensitivity = 1.00, specificity = 0.95), respectively. We also evaluated Miki’s criteria for PG test in diagnosing AIG (Table 3). Sensitivity and specificity of PG 3 + were 85 and 100%, respectively. On the other hand, sensitivity and specificity of PG 2 + or 1 + were 90 and 66.7%, respectively. Among histopathologically confirmed AIG patients, PG I ranged from 2.5 to 71.9 (mean ± SD = 13.3 ± 19.5) ng/mL, PG II from 3.7 to 39.2 (mean ± SD = 10.9 ± 8.3) ng/mL and PG I/II ratio from 0.3 to 3.5 (mean ± SD = 1.0 ± 0.7). PG I, PG II and PG I/II ratio were not statistically different between histopathological stages (Table 4). PG test classification revealed 3 + in 17 (85%), 2 + in 1 (5%), 1 + in 0 (0%) and negative in 2 patients (10%) (Fig. 4). Of these 20 patients, 4 and 16 patients were included in florid and end stages, respectively, but the results of PG test were not related to the stage of AIG.

**Table 3:** Comparison of sensitivity and specificity between criteria for PG test

PG test	Number of patients AIG		Sensitivity (95% CI)	Specificity (95% CI)
	+	−		
3 +	17	0	85 (62.1–96.8)	100 (54.1–100)
2 +, 1 +, −	3	6		
3 +, 2 +	18	2	90 (68.3–98.8)	66.7 (22.3–95.7)
1 +, −	2	4		
3 +, 2 +, 1 +	18	2	90 (68.3–98.8)	66.7 (22.3–95.7)
−	2	4		

All patients were classified into four groups (3 +, 2 +, 1 +and −) according to Miki's criteria

PG, pepsinogen; AIG, autoimmune gastritis; 95% CI, 95% confidence interval

**Table 4:** Comparison of serum PGs between stages of AIG

Serum PGs	Mean ± SD Stage of AIG		*P* value
	Florid stage (N = 4)	End stage (N = 16)	
PG I (ng/mL)	22.7 ± 24.5	11.0 ± 17.3	0.31
PG II (ng/mL)	11.9 ± 4.1	10.6 ± 9.1	0.80
PG I/II ratio	1.5 ± 1.2	0.9 ± 0.5	0.39

Serum PGs were compared between florid and end stages; *t-*test

PG, pepsinogen; SD, standard deviation; AIG, autoimmune gastritis

**Fig. 5:**
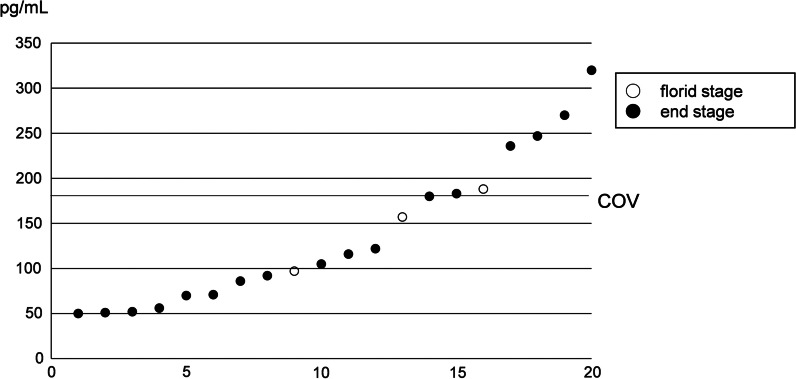
Distribution of serum vitamin B_12_ (N = 20). COV; cut-off value (180 pg/mL). Vitamin-B_12_ users were not included in the figure. The numbers in the horizontal axis are not the patient’s numbers in Table 2. Patients are ordered by the values of serum vitamin B_12_. Thirteen patients (65%) showed low serum vitamin B_12_. Three patients were included in florid stage (○), and 17 patients were included in end stage (●)

**Fig. 6:**
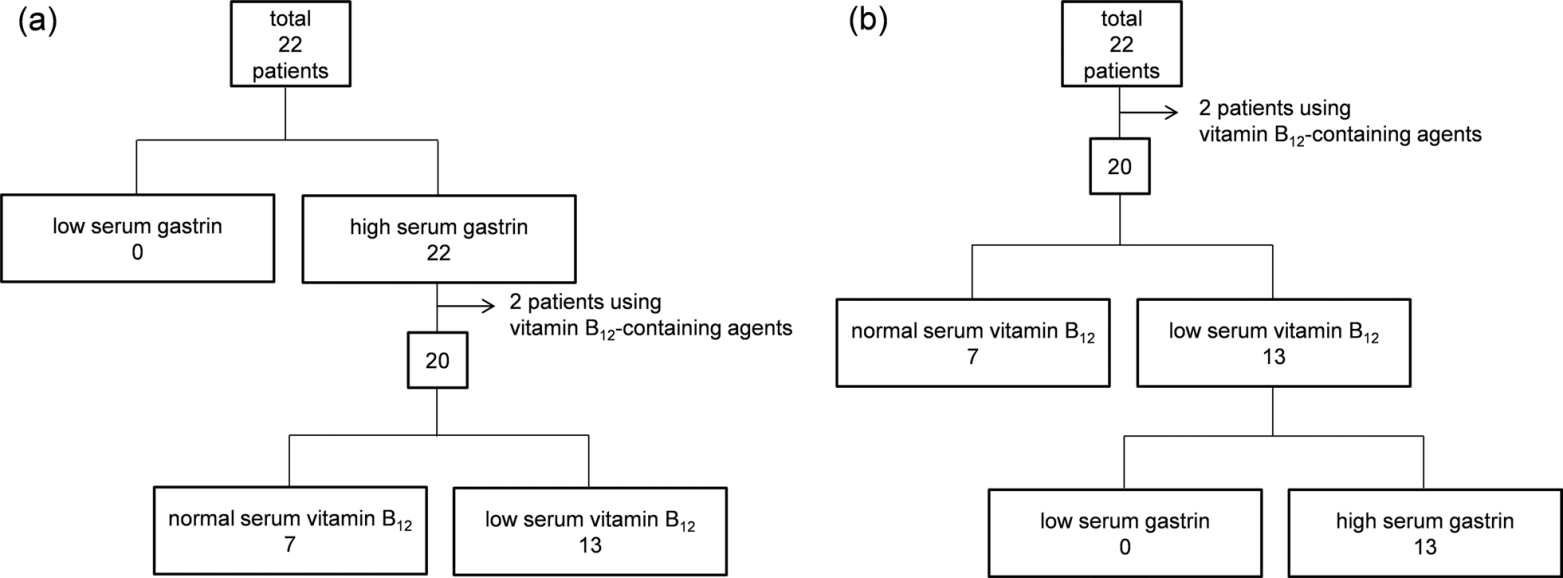
Diagram of AIG patients according to serum gastrin (**a**) and serum vitamin B_12_ (**b**). All the 22 AIG patients showed high serum gastrin. Among them, 20 patients did not use vitamin B_12_-containing agents and 13 patients (65%) of them showed low serum vitamin B_12_ (**a**). In the 20 patients who did not use vitamin B_12_-contaning agents, 13 patients showed low serum vitamin B_12_. All of them showed high serum gastrin (**b**)

Serum vitamin B_12_ was measured in all 22 patients but 2 of them used vitamin B_12_-containing agents and the values were higher than 400 pg/mL. In the rest of 20 patients, vitamin B_12_ ranged from 50 to 320 (mean ± SD = 137.5 ± 79.2) pg/mL (Fig. 5). According to the cut-off value (180 pg/mL), 13 patients (65%) showed low serum vitamin B_12_. Of the 20 patients, 3 and 17 patients were included in florid and end stages, respectively (Fig. 5). Serum vitamin B_12_ was not statistically different between histopathological stages of AIG (florid stage: mean ± SD = 147.3 ± 37.8 pg/mL; end stage: 135.7 ± 84.3 pg/mL; *P* = 0.83, *t*-test). All 22 patients showed high serum gastrin and 20 of them did not use vitamin B_12_-containing agents. Among them, 7 and 13 patients showed normal and low serum vitamin B_12_, respectively (Fig. 6). On the other hand, in the 20 patients without vitamin B_12_ users, 7 and 13 showed normal and low serum vitamin B_12_, respectively. All the patients with low serum vitamin B_12_ showed high serum gastrin (Fig. 6).

Endoscopic corpus atrophy was evaluated in all the 22 AIG patients. Among them, 20 (90.9%), 1 (4.5%) and 1 (4.5%) were classified in O-p, O-3 and O-1, respectively. Therefore, all the 22 AIG patients had endoscopic atrophy O-1 or greater. Of 4 patients in florid stage, 3 were classified in O-p and one in O-3. Of 18 patients in end stage, 17 were classified in O-p and one in O-1. There was no significant difference in O-p ratio between florid and end stage (75% vs 94.4%, respectively, *P* = 0.22, *chi*-square test, Table 5).

**Table 5:** Comparison of endoscopic corpus atrophy between stages of AIG

Endoscopic corpus atrophy	Number of patients Stage of AIG	
	Florid stage (N = 4)	End stage (N = 18)
O-p	3	17
other than O-p	1	1
O-p ratio	75%	94.4%

Endoscopic corpus atrophy was evaluated with Updated Kimura–Takemoto classification

O-p ratio was compared between florid and end stages; *P* = 0.22, *chi*-square test

AIG; autoimmune gastritis

We counted the number of positive items among the 4 tests: elevated serum gastrin, endoscopic O-p atrophy, 3 + PG test and low serum vitamin B_12_. Four items were positive only in 11 AIG patients (50%). On the other hand, the number of positive items was 1 in 1 patient, 2 in 3 patients and 3 in 7 patients, respectively (Table 2). Of the 7 patients whose number of positive items was 3, 6 patients had normal serum vitamin B_12_ and 1 had 2 + PG test. Common clinical findings in the confirmed AIG patients were 10 × or greater anti-PC antibody, elevated serum gastrin greater than 172 pg/mL and endoscopic atrophy O-1 or greater. Other items were not common in all the 22 AIG patients.

## Discussion

In the present study, 22 of 28 (78.6%) patients those who met the screening criteria for candidates were histopathologically confirmed as AIG. Our screening criteria worked well with 78.6% positive predictive value. Currently *H. pylori*-infected patients were not found in any of the 22 AIG patients. We could not find any AIG patients in histopathologically early stage but found 4 in florid and 18 in end stages. We could not find a significant difference between florid and end stages in the items studied. We extracted common clinical findings in the confirmed AIG patients: 10 × or greater anti-PC antibody, elevated serum gastrin greater than 172 pg/mL and endoscopic atrophy O-1 or greater. We found that PG I and PG I/II ratio may be useful to screen AIG according to ROC analyses, and the optimal cut-off values of PG I and PG I/II ratio were 14.5 ng/mL and 2.1, respectively.

We evaluated serum gastrin in AIG patients. In 22 patients who were histopathologically diagnosed AIG, serum gastrin was markedly elevated in both florid and end stages (Table 2). Although serum gastrin was not statistically different between florid and end stages of AIG, those who showed higher serum gastrin than 3000 pg/mL were all in end stage (Fig. 2). These results suggest that gastrin increases with the progress of histopathological AIG. In addition, the normal cut-off value of serum gastrin (172 pg/mL) could also be a suitable cut-off value for screening AIG. Because no patients were classified into early stage and we studied only a small number of patients in florid stage, more numbers of patients are needed to determine the precise cut-off value to screen AIG.

The present study is the first study to evaluate the ability of PG test to screen candidates of AIG. Using Miki’s criteria, 3 + PG test showed high sensitivity and specificity in the present study (Table 3). According to the ROC curves and AUC analyses, PG I and PG I/II ratio can be used for screening AIG (Fig. 3). The optimal cut-off values for PG I and PG I/II ratio were suggested 14.5 ng/mL and 2.1, respectively. These cut-off values can be proposed as criteria for diagnosing AIG, although the precise cut-off values should be determined with more numbers of patients. We also investigated the differences of PGs between histopathological stages (Table 4). However, the PGs and PG I/II ratio were not statistically different between florid and end stages. In addition, PG test evaluation with Miki’s criteria was not related to the histopathological stages (Fig. 4). More numbers of patients may be needed to evaluate PG test in each histopathological stage.

We also included low serum vitamin B_12_ in the screening criteria for AIG. In the present study, 13 patients (65%) showed low serum vitamin B_12_, and all the patients with low serum vitamin B_12_ showed high serum gastrin (Fig. 6). On the contrary, not all patients with high serum gastrin showed low serum vitamin B_12_ (Fig. 6). These facts indicate that serum gastrin exceeds the normal range before serum vitamin B_12_ falls. In other words, serum gastrin elevates first and vitamin B_12_ falls later in the progress of AIG. Therefore, low serum vitamin B_12_ may be a marker of severe fundic atrophy in AIG, but we could not obtain clear evidence for the relationship between serum vitamin B_12_ and the histopathological stages in the present study (Fig. 5).

We evaluated endoscopic corpus atrophy in all the 22 AIG patients with Updated Kimura–Takemoto classification [7, 8]. Among them, 21 (95.5%) were classified in O-p or O-3, and one patient was classified in O-1 atrophy. This fact suggests that AIG is strongly suspected in patients classified in O-p or O-3. The common finding of endoscopic atrophy was at least O-1 atrophy. Because there was not a significant difference in O-p ratio between florid and end stages, endoscopic corpus atrophy was not always related to the histopathological stages (Table 5). The discrepancy between endoscopic and histopathological atrophy may be due to the difference between macroscopical and microscopical points of view. Endoscopic diagnosis of atrophy is a lateral overall diagnosis including mucosa and deeper layers whereas histopathological diagnosis from biopsy samples is a point diagnosis of the surface part of gastric wall. In addition, histopathological diagnosis is largely affected with the part where biopsy specimen was taken. It is reported that remnant fundic glands exist in some AIG patients [16], so that gastric atrophy may not be diagnosed microscopically if the biopsy sample was taken from the remnant fundic mucosa. These may be the possible reasons why histopathological stages were not related to endoscopic atrophy.

We screened AIG with autoantibodies and four non-invasive tests in the present study. The number of positive items in the latter four tests ranged from 1 to 4 (Table 2). The only common item in the AIG patients examined was elevated serum gastrin. It suggests that elevated serum gastrin may be essential to diagnose AIG and may be enough for the screening. The cut-off value of manufacturer’s instruction (172 pg/mL) was suitable in the subjects of this study. Because patients with renal dysfunction or using PPI tend to have elevated serum gastrin [17, 18], it is not suitable to use serum gastrin to screen AIG in such patients. In addition, endoscopic findings and PG tests also tend to show abnormal in PPI users [19, 20], so that serum vitamin B_12_ test may be an only trustable test to screen AIG in such patients. However, serum vitamin B_12_ was decreased in only 13 of 20 AIG patients (65%) (Fig. 5), and those who take vitamin B_12_-containing agents had an increased serum B_12_, it may not be enough to screen AIG with single serum vitamin B_12_ test, but adding vitamin B_12_ test may be recommended.

No patients were histopathologically confirmed as early stage of AIG in the present study. On the other hand, all AIG patients in the present study were either in florid or end stages. Early stage of AIG may be difficult to screen with our criteria. We could not find significant differences between florid and end stages in serum gastrin, PGs, vitamin B_12_ or endoscopic atrophy in the present study. These facts may indicate that there is no major clinical difference between florid and end stages and suggest that florid stage is already an advanced stage of AIG in clinical aspects. Our criteria may be able to screen only advanced stages of AIG. We may need to find other criteria to screen early stage of AIG.

The present study had some limitations. First, this was a retrospective observational study performed at a single institution. More numbers of patients are needed to determine the power of the screening criteria and to find suitable cut-off values to screen AIG. Second, we could not test anti-IF antibody in all the patients with negative anti-PC antibody test, because the test was out of insurance coverage in Japan and expensive. Because the sensitivity and specificity of anti-PC antibody are reported to be 81% and 90%, respectively [21], we may have missed some AIG patients. It is expected that the combination of anti-PC and anti-IF antibody tests increase the diagnostic performance in the future [21]. Third, the relation between *H. pylori* infection and AIG remains unclear. We did not diagnose AIG with current *H. pylori* infection because it is difficult to discriminate *H. pylori*-infected gastritis alone from *H. pylori*-infected AIG. Although we mentioned that we had 12 patients with previous *H. pylori* infection, only 7 patients had history of receiving *H. pylori* eradication therapy, but the remaining did not have such history but suspected as past infection by medical doctors. In addition, it is unknown whether there was clear evidence of *H. pylori* infection before eradication therapy because it is reported that patients with AIG often show false positive results in non-invasive *H. pylori* tests [22]. It means that the diagnosis of past infection may have been wrong in some patients, and patients with past *H. pylori* infection may have been contaminated with never-infected patients. For these reasons, we could not mention about the relation between *H. pylori* infection and AIG. At least we can say that considerable number of patients could be diagnosed as AIG within four years after eradication therapy. The relation between *H. pylori* infection and AIG should be studied with strictly diagnosed patients for *H. pylori* in the future.

## Conclusions

Florid stage in histopathology may be already in an advanced stage of AIG in clinical aspects as well as end stage. Our screening criteria without biopsy are applicable to screen clinically advanced AIG with 78.6% positive predictive value. PG I and PG I/II ratio may be useful to screen AIG, and the optimal cut-off values of PG I and PG I/II ratio were 14.5 ng/mL and 2.1, respectively. However, we may need other criteria to screen early stage of AIG.

## Data Availability

The datasets during and/or analyzed during the current study available from the corresponding author on reasonable request.

## Abbreviations

AIG: Autoimmune gastritis
PC: Parietal cell
IF: Intrinsic factor
PG: Pepsinogen
H&E: Hematoxylin and eosin
ECL: Enterochromaffin cell-like
*H**. pylori*: *Helicobacter pylori*
HpAb: *H. pylori* Antibody
HpSA: *H. pylori* Stool antigen
UBT: Urea breath test
PPI: Proton pump inhibitor
ROC: Receiver operating characteristic
AUC: Areas under the curve
